# Evaluation of the recovery after heart surgery following preoperative supplementation with a combination of beta-hydroxy-beta-methylbutyrate, l-arginine, and l-glutamine: a double-blind randomized placebo-controlled clinical trial

**DOI:** 10.1186/s13063-022-06621-1

**Published:** 2022-08-13

**Authors:** Mona Norouzi, Azadeh Nadjarzadeh, Majid Maleki, Sayyed Saeid Khayyatzadeh, Saeid Hosseini, Mehdi Yaseri, Hamed Fattahi

**Affiliations:** 1 Nutrition and Food Security Research Center, Shahid Sadoughi University of Medical Sciences, Shohadaye gomnam BLD., ALEM square, Yazd, Iran; 2 Department of Nutrition, International Campus of Shahid Sadoughi University of Medical Sciences, Yazd, Iran; 3 Department of Nutrition, Faculty of Health, Shahid Sadoughi University of Medical Sciences, Yazd, Iran; 4 Rajaie Cardiovascular Medical and Research Center, Iran University of Medical Sciences, Tehran, Iran; 5 Heart Valve Disease Research Center, Shahid Rajaie Cardiovascular Medical and Research Center, Iran University of Medical Sciences, Tehran, Iran; 6 Department of Epidemiology and Biostatistics, Tehran University of Medical Science, Tehran, Iran; 7 Cardiovascular Medical and Research Center, Shahid Beheshti University of Medical Sciences, Tehran, Iran

**Keywords:** Beta-hydroxy-beta-methylbutyrate, Glutamine, HMB, Arginine, Heart surgery, Recovery

## Abstract

**Background:**

The preoperative period is a good time to improve nutrition status, compensate for nutrient deficiencies, and optimize immune function in patients’ underlying surgery. In some medical conditions, supplementation with a combination of l-glutamine (Gln), β-hydroxy-β-methylbutyrate (HMB), and l-arginine (Arg) had promising effects on improving recovery. The present study aimed to evaluate the effect of supplementation with Gln/Arg/HMB in patients undergoing heart surgery.

**Methods:**

This randomized clinical trial was conducted on 70 patients undergoing cardiac surgery. Participants were requested to consume 2 sachets of a combination of 7 g l-arginine, 7 g l-glutamine, and 1.5 g daily HMB or placebo 30 days before operation. At the baseline and end of the study, left ventricular ejection fraction and the serum levels of troponin, creatine phosphokinase (CPK), CPK-MB, alanine aminotransferase (ALT), aspartate aminotransferase (AST), and bilirubin were measured. Also, the Sequential Organ Failure Assessment (SOFA) score, time of stay in hospital and intensive care unit (ICU), and postoperative complications were recorded after surgery.

**Results:**

In total, 60 preoperative patients (30 in each group) with a mean age of 53.13 ± 14.35 years completed the study (attrition rate = 85.7%). Subjects in the Gln/Arg/HMB group had lower serum levels of CPK-MB (median [IQR] = 49 [39.75] vs. 83 [64.55]; *P* = 0.011), troponin (median [IQR] = 2.13 [1.89] vs. 4.34 [1.99]; *P* < 0.001), bilirubin (median [IQR] = 0.50 [0.20] vs. 0.40 [0.22]; *P* < 0.001), and SOFA score (median [IQR] = 2 [2] vs. 5 [2]; *P* < 0.001) at end of the study compared to the placebo. Also, the time of stay in the hospital (median [IQR] = 5 [1] vs. 6 [3]; *P* < 0.001) and ICU (median [IQR] = 2.50 [1.00] vs. 3.50 [1.50]; *P* = 0.002) was lower in the Gln/Arg/HMB group.

**Conclusion:**

The present study showed that perioperative supplementation with a combination of Gln, Arg, and HMB enhances the recovery, reduces myocardial injury, and decreases the time of hospital and ICU stay in cardiac surgery patients. These results need to be confirmed in a larger trial.

**Trial registration:**

IRCT.ir IRCT20120913010826N31. Registered on 13 October 2020.

## Introduction

In recent years, the aging population has resulted in a significant increase in cardiac operations, as is estimated to occur at about 2 million per year worldwide [1]. Despite advances in this area, there are still complications as a result of complex cardiosurgical procedures that are associated with an increase in morbidity and mortality [2]. It has been demonstrated that poor nutritional status affects outcomes following surgery. Nutritional deficiencies may induce inflammation by reducing the defense system function and depleting antioxidant nutrient reserves [3]. At the other hand, some studies reported a drop in serum amino acid levels such as citrulline, arginine (Arg), and glutamine (Gln) after cardiac surgery [4]. It has been reported that the depletion of micronutrients and macronutrients in people undergoing heart surgery makes them more susceptible to sarcopenia, cachexia, surgical trauma, hemodilution, pneumonia, and infection. The result is a longer recovery process and increased length of stay in the intensive care unit [3, 5].

The nutritional status of the patients during surgery is less considered in clinical practice. Delayed initiation of nutritional support in heart surgery may be associated with an increased risk of iatrogenic malnutrition [6]. Although there are recommendations for nutritional support immediately after surgery, evidence for preoperative nutrition is limited [7]. The preoperative period is a good time to improve nutrition status, compensate for nutrient deficiencies, and optimize immune function [6]. In cardiac patients, it has been revealed that fasting before the surgery aggravates dyspnea, gut edema, hepatic congestion, and nausea [6, 8]. Moreover, systemic inflammation results in protein-calorie malnutrition that is associated with weakness, poor recovery and wound healing, and morbidity after surgery [9]. It is generally agreed that optimized medical and nutritional status before surgery can help the patient reduce the stress of surgery, maintain the physiological hemostasis, and enhance recovery [6]. However, limited information on the principles of preoperative care necessitates further studies.

In previous studies, the effect of protein supplements has been investigated in patients subjected to cardiac surgery [6]. Although some improvements were indicated in the recovery and modulating the inflammatory responses following high-protein intake [10], other studies suggest a restriction in total protein intake and focus on single amino acids [11]. It has been revealed that the intake of some specific amino acids may be more effective in the enhancement of the recovery process [12]. Also, previous studies showed a decrease in the mortality rate of critically ill patients following high-protein nutritional support enriched with immune-modulating nutrients, including Gln, omega-3 fatty acids, selenium, and antioxidants [13]. Gln and Arg are two amino acids with cardioprotective effects that have recently received a lot of attention. Studies showed that preoperative supplementation of Gln reduces myocardial injury in cardiac surgery patients [14]. Arg exerts its positive effects on the cardiovascular system through the production of nitric oxide, which increases blood flow and improves vascular damage [15]. Moreover, there are reports of proinflammatory cytokine downregulation following Gln and Arg co-administration in other types of major surgery [16]. The β-hydroxy β-methylbutyric acid (HMB), a metabolite of leucine, is another nutrient that is involved in the synthesis of muscle protein and in reducing protein catabolism [17]. In addition, supplementation with these three amino acids is effective in wound healing, preventing cachexia, and increasing muscle mass in hypercatabolic individuals [18]. In a clinical trial study, supplementation with a combination of Gln/Arg/HMB 3 days before and 7 days after abdominal surgery failed to improve the wound healing. However, a positive increase was observed in hormones related to recovery, such as serum growth hormone levels [19]. In a study with a longer intervention period (20 days), a significant change in wound healing and biochemical factors was observed in patients with pressure ulcers [20]. Despite this evidence, guidelines have not recommended the routine use of these supplements due to insufficient studies. Further studies are required to prove the clinical benefits of these supplements in different groups and with longer duration of intervention. The present study aimed to evaluate the effect of HMB/Arg/Gln co-supplementation in 1 month before cardiac surgery on postoperative recovery.

## Materials and methods

### Study design and participants

This study was conducted in a double-blind randomized clinical trial design in candidates for cardiac surgery referred to Shahid Rajaei educational, research, and medical center of cardiovascular diseases, Tehran, Iran, in 2021. A total of 70 patients participated, with a 1:1 ratio of allocation to the intervention and control groups in parallel method. The following equation was used to calculate the sample size [21]:

$$n=\frac{2\times {\left({Z}_{1-\frac{\alpha }{2}}+{Z}_{1-\beta}\right)}^2\times \left(2{\sigma_{\mathrm{diff}}}^2\right)\ }{\updelta^2}$$

According to a pilot study on 50 patients, *δ* was considered equal to the 1.8 unit difference of changes in serum troponin between groups (*σ*_diff_^2^ = 1.98). By considering 0.05 probability of type I error (*α*), 95% of power (1−*β*), and 10% of drop-out, the sample size was determined by 35 subjects in each group.

Inclusion criteria for the study were the age between 18 and 70 years, body mass index (BMI) between 18.5 and 29.9 kg/m^2^, and planning to perform one of the cardiosurgical procedures. Patients with mitral valve replacement, aortic root surgery, aortic valve replacement, coronary artery bypass graft surgery, or a combination of these for the first time were eligible for the study. The exclusion criteria were the history of previous cardiac surgeries; the presence of infection; sepsis; allergies; smoking; drug or alcohol abuse; history of kidney diseases; diabetes mellitus; autoimmune diseases; cancer; dysfunction of the thyroid, pituitary, and hypothalamus; gastrointestinal diseases; and hepatic disease. Patients who have conditions that require the use of other enteral formulas or those receiving immunosuppressive and metabolism-modulating drugs were also excluded from the study. The aims of the study were described to the patients and those interested in participating in the study signed a written informed consent. It was planned to exclude patients in case of low compliance, emergency cardiac surgery before 1 month of intervention, and manifestation of adverse effects related to the supplement or placebo intake. The Declaration of Helsinki was used to design and conduct the study. Also, the protocol of the study was approved by the Ethics Committee of Shahid Sadoughi University of Medical Sciences (IR.55U.SPH.REC.1398.122). The protocol of the study was registered in the Iranian Registry of Clinical Trials with registration no. IRCT20120913010826N31 at 2020-10-13.

### Randomization, blinding, and intervention

**Fig. 1:**
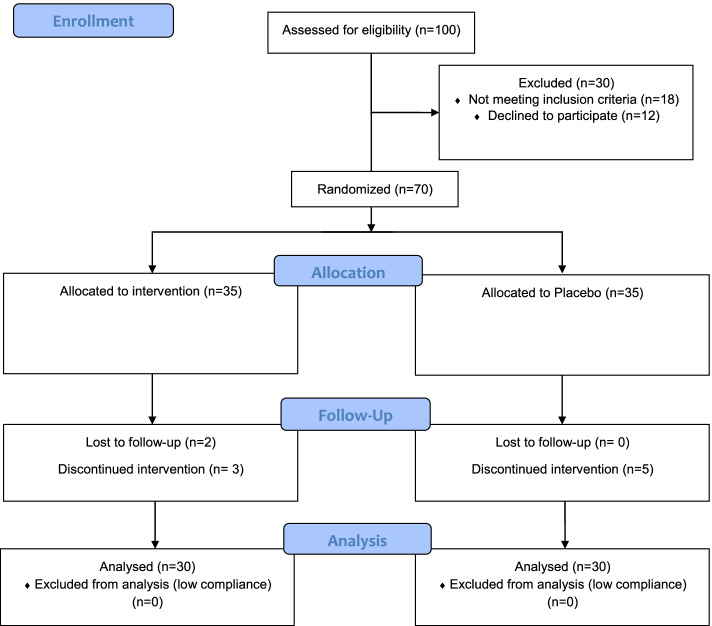
The CONSORT flow diagram of the study participants

Figure 1 depicts the CONSORT flowchart of participants’ enrollment. Sampling was performed by the block randomization method. The block size was 2 and subjects were allocated randomly to the intervention or placebo groups by a person blinded to the aims of the study using sealed envelopes. Also, researchers and study subjects were blinded to the allocation sequence. Subjects in the intervention group received 2 sachets/day of a combination of 7 g l-arginine, 7 g l-glutamine, and 1.5 g HMB (Heallagen®) for 1 month before the operation. Patients assigned to the placebo group received 2 sachets/day of maltodextrin with identical taste and appearance to Heallagen®. Both the Heallagen® and placebo were manufactured by Karen Pharma & Food Supplements company (Tehran, Iran). Routine medications that were formerly prescribed for the subject’s heart condition were continued and they were asked not to receive any other medication or dietary supplements.

### Procedures

The study started 30 days before cardiac surgery. After the assignment, participants received 60 sachets of Gln/Arg/HMB or placebo and they were asked to intake 2 sachets with 120 cc of water each day. To evaluate the participants’ compliance, they were requested to return the package of used sachets on the day before surgery. Patients who consumed less than 54 sachets (90%) were excluded from the study due to low compliance. Also, subjects in both groups were called every week to record the occurrence of any adverse effect. The general characteristics including age, sex, history of disease, medication and supplement use, history of hypertension (HTN), weight, height, and the use of tobacco were recorded at the start of the study (Table 1). Also, venous blood samples were collected at the baseline and 1 day after surgery for biochemical assessment. Moreover, at the end of the study, the postoperative recovery of patients was recorded. Confidentiality of participants’ personal information was protected and every subject was specified by an identification number throughout the study.

**Table 1:** Baseline characteristics of the participants according to the Gln/Arg/HMB or placebo groups^1^

Variable	Gln/Arg/HMB (*n* = 30)	Placebo (*n* = 30)	*P*^2^
Age (years)	59.00 (29.50)	55.00 (14.50)	0.706*
Height (cm)	169.23 ± 9.69	168.47 ± 8.40	0.319
Weight (kg)	73.50 (12.50)	75.00 (12.50)	0.772*
Body mass index (kg/m^2^)	25.25 ± 4.34	26.30 ± 4.61	0.364
Gender			
Male	21 (70.0)	15 (50.0)	0.114
Female	9 (30.0)	15 (50.0)	
Hypertension history			
Yes	0 (0.0)	3 (10.0)	0.236
No	30 (100.0)	27 (90.0)	

^1^Data are presented as mean ± SD or median (IQR) for quantitative variables and frequency (%) for qualitative variables

^2^Calculated using the independent sample *t*-test or Mann-Whitney *U*-test (indicated by “*”) for quantitative variables and chi-square for qualitative variables

### Place of the study and competency of investigators

The study was conducted in Shahid Rajaei educational, research, and medical center of cardiovascular diseases, Tehran, Iran. This center is one of the largest heart hospitals in the Middle East, which includes the operating room, catheterism and angiography, radiology, central laboratory, medical research laboratory, experimental research laboratory, dialysis ward, physiotherapy, autopsy and cardiovascular research center. The research team consisted of a cardiologist, a heart surgeon, 2 nutritionists, a nurse, a laboratory specialist, and a statistical consultant who were responsible for preoperative and postoperative visits, patient screening, surgery, patient nutritional evaluation, proper supplementation, statistical analysis, and conducting a pilot study to estimate sample size.

### Primary outcomes

Recovery after heart surgery was measured using the levels of troponin, creatine phosphokinase (CPK), and creatine phosphokinase myocardial band (CPK-MB); left ventricular ejection fraction (LVEF); and Sequential Organ Failure Assessment (SOFA) score. LVEF was estimated using a 3-dimensional (3D) transthoracic echocardiography (Philips ie33, Koninklijke Philips Electronics, Amsterdam, the Netherlands) with standard protocols by an experienced cardiologist [22]. The SOFA score is a checklist that involves the function of the pulmonary, cardiovascular, hepatic, renal, and central nervous systems [23]. This instrument evaluates the condition of the body organs systematically and continuously during the patient’s hospitalization in the intensive care unit (ICU).

### Secondary outcomes

Secondary outcomes that were evaluated in the present study are grouped as follows.

#### Hepatic enzymes

The serum levels of alanine aminotransferase (ALT), aspartate aminotransferase (AST), and bilirubin (total, direct, and indirect) were measured to compare hepatic enzymes between the two groups.

#### Stay in hospital and ICU

The time of stay in the hospital and ICU was recorded for all participants. Discharge from the hospital was defined when patients fulfilled all the following criteria: stable heart rhythm, ability to tolerate oral solutions and solid foods, mobility without assistance, normal range of temperature, normal urination and defecation, normal wound healing, no complications requiring hospital stay, normal laboratory data, and a proper heart function. These evaluations were performed by an independent surgeon who was not aware of the research protocol.

#### Start of food intake, postoperative complications, and nutritional risk

The start of liquid and solid food intake and complications after surgery were recorded. Complications after surgery included the occurrence of myocardial infarction (MI), stroke, acute kidney injury (AKI), atrial fibrillation (AF), coma, and acute respiratory distress syndrome (ARDS) that were constantly monitored by a cardiologist and recorded in the patients’ file. Nutritional risk of patients were assessed using the Modified NUTrition Risk in Critically ill (mNUTRIC) score after surgery that was translated and validated specifically for ICU patients to use in Iran [24].

### Biochemical assessments

At the start of the study and 1 day after surgery, 5 ml of antecubital venous blood samples was collected between 7:00 and 9:00 am to perform biochemical analysis. First, samples were centrifuged for 10 min at room temperature at 4000 rpm to isolate the serum. Then, the serum levels of CPK, CPK-MB, ALT, AST, and bilirubin were assessed using commercial kits (Pars Azmun, Karaj, Iran) and Hitachi 917 autoanalyzer (Japan). The troponin level was measured using the highly sensitive troponin immunoassay (Abbott) method.

### Anthropometric measurements

The weight of participants was assessed using a Seca scale with a precision of 100 g. The height was measured using a Seca stadiometer to the nearest 1 mm. In case of inability to stand, a tape measure was used to evaluate the length. Measurements were repeated 3 times with minimal clothes, without shoes, and after overnight fasting. BMI was calculated as weight (kg) divided by the square of height (m^2^).

### Statistical methods

The Shapiro-Wilk test was used to evaluate the normality of continuous variables. The normally distributed variables were presented as mean ± SD. Non-normal variables were reported as median (IQR). If a log-transformed variable had a normal distribution, its log was used in the analysis. The qualitative variables were reported as frequency (%). To compare groups regarding these variables (gender, history of hypertension, and presence of nausea, vomiting, and complications after surgery), the chi-square test was used. The continuous variables (age, anthropometric measures, myocardial biomarkers, LVEF, SOFA score, hepatic enzymes, duration of hospital residence, and time of food intake after surgery) were compared between two groups via the independent sample *t*-test or Mann-Whitney *U*-test. To assess the within-group change in continuous variables, the paired sample *t*-test or Wilcoxon test was performed. To adjust the effect of confounding variables, BMI, age, sex, and history of HTN were entered into the analysis of covariance (ANCOVA) or generalized estimating equation (GEE) for normally and non-normally distributed or binomial variables, respectively. The SPSS software version 25 (IBM Corp. IBM SPSS Statistics for Windows, Armonk, NY) was used to perform statistical analyses. The *P*-value < 0.05 was considered statistically significant.

## Results

### Study general characteristics

In the present study, 70 patients were assigned to the Gln/Arg/HMB or placebo groups from January to December 2020. In total, 8 subjects (3 in the Gln/Arg/HMB group and 5 in the placebo group) discontinued to participate in the study. Also, 2 subjects in the Gln/Arg/HMB group lost to follow-up. Sixty subjects (30 in the Gln/Arg/HMB and 30 in the placebo group) with a mean age of 53.13 ± 14.35 and BMI equal to 25.78 ± 4.48 completed the study (attrition rate = 85.7%). Consumption of the sachets was more than 90% in all participants and no adverse effect was reported. More than half of the participants were male (60%), 3 of them (5%) had a history of HTN. Preliminary comparison between the two groups found no difference in age (*P* = 0.706), weight (*P* = 0.772), BMI (*P* = 0.364), gender (*P* = 0.114), and history of HTN (*P* = 0.236).

### Primary outcomes

Table 2 outlines the myocardial markers and postoperative recovery of the participants at the baseline and end of the study. At the baseline, no significant difference was observed in the serum concentration of CPK, CPK-MB, and troponin (*P* > 0.05). The paired sample *t*-test or Wilcoxon rank test showed a significant increase in CPK (median [IQR] = 123 [295.75] vs. 336.5 [271.75], respectively), CPK-MB (median [IQR] = 28.50 [42.3] vs. 64.80 [60.32], respectively), and troponin (median [IQR] = 2.1 [1.9] vs. 4.32 [2.01], respectively) in the intervention and placebo groups (*P* < 0.001). The changes in CPK-MB and troponin were greater in the placebo group compared to the Gln/Arg/HMB (*P* = 0.008 and *P* < 0.001, respectively). At the end of the study, the Gln/Arg/HMB group had lower levels of CPK-MB (median [IQR] = 49 [39.75] vs. 83 [64.55]; *P* = 0.011) and troponin (median [IQR] = 2.13 [1.89] vs. 4.34 [1.99]; *P* < 0.001) compared to the placebo. No significant difference was observed between the two groups in CPK levels at the end of the study (*P* = 0.121). Adjustments for the effect of BMI, age, gender, and history of hypertension did not change these results.

**Table 2:** Comparison of the myocardial biomarkers and postoperative recovery between groups at the baseline and end of the study^1^

Variable^2^	Gln/Arg/HMB (*n*=30)	Placebo (*n*=30)	*P*^3^	*P*-adjusted 1^4^
CPK (μg/L)				
Baseline	89.00 (70.25)	65.50 (117.0)	0.610*	0.563^¥^
End of the study	370.40 ± 447.40	396.73 ± 176.76	0.121**	0.156**
Changes	123.0 (295.75)	336.50 (271.75)	0.059*	0.116^¥^
*P*^5^	<0.001*	<0.001*		
CPK-MB (IU/L)				
Baseline	19.45 (5.80)	18.00 (5.25)	0.858*	0.526^¥^
End of the study	49.00 (39.75)	83.00 (64.55)	0.011*	0.032^¥^
Changes	28.50 (42.3)	64.80 (60.32)	0.008*	0.036^¥^
*P*^5^	<0.001*	<0.001*		
Troponin (ng/mL)				
Baseline	0.02 (0.03)	0.02 (0.02)	0.512*	0.575^¥^
End of the study	2.13 (1.89)	4.34 (1.99)	<0.001*	<0.001^¥^
Changes	2.10 (1.9)	4.32 (2.01)	<0.001*	<0.001^¥^
*P*^5^	<0.001*	<0.001*		
LVEF (%)				
Baseline	55.0 (5.0)	50.0 (5.0)	0.021*	0.036^¥^
End of the study	55.0 (5.0)	50.0 (6.25)	0.105*	0.088^¥^
Changes	0.0 (0.0)	0.0 (0.0)	0.531*	0.313^¥^
*P*^5^	0.157*	0.317*		
SOFA score	2.00 (2.00)	5.00 (2.00)	<0.001*	<0.001^¥^

^1^*CPK* creatine phosphokinase, *CPK-MB* creatine phosphokinase myocardial band, *LVEF* left ventricular ejection fraction, *SOFA* Sequential Organ Failure Assessment

^2^Data are presented as mean ± SD for normally distributed continuous variables or as median (IQR) for non-normally distributed continuous variables

^3^Calculated using the independent sample *t*-test (variables that entered as logarithm form are indicated by “**”) or Mann-Whitney *U*-test (indicated by “*”)

^4^Calculated using ANCOVA (variables that entered as logarithm form are indicated by “**”) or non-parametric ranked ANCOVA using generalized estimation equation (indicated by “^¥”^), adjusted for the effect of BMI, age, gender, and history of HTN

^5^Calculated using the paired *t*-test or Wilcoxon rank test (indicated by “*”)

At the start of the study, the Gln/Arg/HMB group had a higher LVEF compared to the placebo group (median [IQR] = 55 [5] vs. 50 [5]; *P* = 0.021). However, at the end of the study, this difference was not statistically significant between the two groups (*P* = 0.105). After surgery, the SOFA score was lower in the Gln/Arg/HMB (median [IQR] = 2 [2]) group compared to the placebo (median [IQR] = 5 [2]; *P* < 0.001). The significance of these differences was not changed after adjustment for the effect of confounding variables.

### Secondary outcomes

#### Hepatic enzymes

As shown in Table 3, at the beginning of the study, subjects in the Gln/Arg/HMB group had a higher total (*P* < 0.001) and indirect (*P* = 0.001) bilirubin compared to the placebo. There was no significant difference between groups in ALT (*P* = 0.960), AST (*P* = 0.246), and direct bilirubin (*P* = 0.065) before the study. Following 1 month of intervention, there was an increase in AST (mean ± SD = 17.83 ± 12.45; *P* < 0.001) and a decrease in total bilirubin (median [IQR] = −0.40 [0.92]; *P* = 0.002) of the Gln/Arg/HMB group. Also, a significant increase was observed in the AST (mean ± SD = 7.30 ± 13.62; *P* = 0.006) and total (median [IQR] = 0.42 [0.60]; *P* = 0.001) and indirect bilirubin (median [IQR] = 0.20 [0.50]; *P* = 0.007) of the placebo group during the study. At the end of the study, the Gln/Arg/HMB group had a higher serum level of AST (mean ± SD = 40.00 ± 14.65 vs. 27.83 ± 13.54; *P* = 0.002) and direct bilirubin (median [IQR] = 0.50 [0.20] vs. 0.40 [0.22]; *P* = 0.004) compared to the placebo. However, the last difference disappeared after adjusting for the effect of confounding variables (*P* = 0.165).

**Table 3:** Comparison of the hepatic biomarkers between groups at the baseline and end of the study^1^

Variable^2^	Gln/Arg/HMB (*n* = 30)	Placebo (*n* = 30)	*P*^3^	*P*-adjusted 1^4^
AST (IU/L)				
Baseline	22.16 ± 4.86	20.53 ± 5.00	0.209	0.246
End of the study	40.00 ± 14.65	27.83 ± 13.54	0.002	0.008
Changes	17.83 ± 12.45	7.30 ± 13.62	0.003	0.011
*P*^5^	<0.001	0.006		
ALT (IU/L)				
Baseline	20.0 (8.0)	21.0 (9.25)	0.450*	0.960^¥^
End of the study	16.0 (13.0)	19.50 (20.50)	0.278*	0.070^¥^
Changes	0.0 (12.5)	-1.50 (20.50)	0.462*	0.145^¥^
*P*^5^	0.123*	0.802*		
Total bilirubin (mg/dL)				
Baseline	1.20 (1.275)	0.50 (0.50)	<0.001*	<0.001^¥^
End of the study	0.80 (0.55)	1.00 (0.50)	0.198*	0.061^¥^
Changes	−0.40 (0.92)	0.42 (0.60)	<0.001*	<0.001^¥^
*P*^5^	0.002*	0.001*		
Direct bilirubin (mg/dL)				
Baseline	0.50 (0.10)	0.50 (0.12)	0.065*	0.024^¥^
End of the study	0.50 (0.20)	0.40 (0.22)	0.004*	0.165^¥^
Changes	0.0 (0.30)	0.0 (0.31)	0.451*	0.965^¥^
*P*^5^	0.301*	0.075*		
Indirect bilirubin (mg/dL)				
Baseline	0.70 (0.50)	0.50 (0.22)	0.003*	0.001^¥^
End of the study	0.60 (0.60)	0.65 (0.50)	0.964*	0.257^¥^
Changes	−0.10 (0.40)	0.20 (0.50)	0.008*	0.012^¥^
*P*^5^	0.214*	0.007*		

^1^*CPK* creatine phosphokinase, *CPK-MB* creatine phosphokinase myocardial band, *AST* aspartate aminotransferase, *ALT* alanine aminotransferase

^2^Data are presented as mean ± SD for normally distributed continuous variables or as median (IQR) for non-normally distributed continuous variables

^3^Calculated using the independent sample *t*-test (variables that entered as logarithm form are indicated by “**”) or Mann-Whitney *U*-test (indicated by “*”)

^4^Calculated using ANCOVA (variables that entered as logarithm form are indicated by “**”) or non-parametric ranked ANCOVA using generalized estimation equation (indicated by “^¥”^), adjusted for the effect of BMI, age, gender, and history of HTN

^5^Calculated using the paired *t*-test or Wilcoxon rank test (indicated by “*”)

#### Start of food intake, postoperative complications, and nutritional risk

Table 4 shows that the nutritional risk was not different between the two groups. Moreover, there was no difference between the groups in the time of NPO. It was found that individuals in the intervention group started receiving food, either fluid (median [IQR] = 6 [0] vs. 9 [4]) or solid (median [IQR] = 8 [0] vs. 11 [2]), earlier than the placebo group (*P* < 0.001). No significant difference was observed in the frequency of nausea (*P* = 0.212), vomiting (*P* = 0.127), and complications after surgery (*P* = 0.236) between the two groups.

**Table 4:** Comparison of hospital residence and food intake after surgery between groups^1^

Variable^2^	Gln/Arg/HMB (*n* = 30)	Placebo (*n* = 30)	*P*^3^	*P*-adjusted^4^
NUTRIC score	3.00 (0.0)	3.00 (1.0)	0.859	0.369
Time of NPO (days)	6.00 (0.0)	6.00 (0.0)	0.078	0.132
Time of fluid food intake (days)	6.00 (0.0)	9.00 (4.00)	<0.001	<0.001
Time of solid food intake (days)	8.00 (0.0)	11.00 (2.00)	<0.001	<0.001
Time of stay in ICU (days)	2.50 (1.00)	3.50 (1.50)	0.001	0.002
Time of stay in hospital (days)	5.00 (1.00)	6.00 (3.00)	<0.001	<0.001
Nausea				
Yes	2 (6.7)	5 (16.7)	0.212	0.116
No	28 (93.3)	25 (83.3)		
Vomiting				
Yes	2 (6.7)	6 (20.0)	0.127	0.054
No	28 (93.3)	24 (80.0)		
Complications after surgery				
Yes	3 (10.0)	6 (20.0)	0.236	0.322
No	27 (90.0)	24 (80.0)		

^1^*NPO* nothing per oral, *ICU* intensive care unit

^2^Data are presented as median (IQR)

^3^Calculated using the Mann-Whitney *U*-test

^4^Calculated using generalized estimation equation (indicated by “^¥^”), adjusted for the effect of BMI, age, gender, and history of HTN

#### Stay in hospital and Intensive Care Unit (ICU)

As shown in Table 4, the Gln/Arg/HMB group spent less time in the ICU (median [IQR] = 2.50 [1.00] vs. 3.50 [1.50]; *P* = 0.001) and was discharged earlier from the hospital (median [IQR] = 5 [1] vs. 6 [3]; *P* < 0.001) compared to the placebo.

## Discussion

The present study showed that the preoperative supplementation with a combination of Gln/Arg/HMB reduces the CPK-MB, troponin, SOFA score, and the time of food intake, stay in the hospital, and ICU residence after cardiac surgery compared to the placebo. In addition, there was an increase in AST and a decrease in total and indirect bilirubin following Gln/Arg/HMB supplementation. No change was observed in total CPK, LVEF, ALT, direct bilirubin, nutritional risk, and incidence of nausea, vomiting, and complications after surgery in the Gln/Arg/HMB group compared to the placebo.

In the present study, preoperative supplementation of Gln/Arg/HMB (14/14/3 g/day, respectively) for 1 month improved recovery and reduced the duration of stay in hospital and ICU, the time to start food intake, and markers of myocardial injury (troponin and CPK-MB) after cardiac surgery. The nutritional risk and LVEF were not different after supplementation between the two groups. However, these variables were in their normal range and no improvement was expected [24, 25]. According to the Society of Thoracic Surgeons (STS), major complications of cardiac surgery include stroke, renal failure, prolonged intubation, unplanned reoperation, and wound infection [26]. Therefore, reducing these factors can help in achieving a successful operation. Although limited studies have examined the combination of these three amino acids for postoperative recovery, there are some reports of their beneficial effects on other medical conditions. In the study of Erdem et al. [27], 28 days of nutritional support with Gln/Arg/HMB (14/14/3 g/day, respectively) among major burn patients caused a significant reduction in the CPK levels. In the study of Wada et al. [19], 3 days preoperative and 7 days postoperative supplementation of Gln/Arg/HMB (7/7/1.2 g/day, respectively) did not change the wound complications but a significant increase in the serum growth hormone (GH) level was observed in subjects with high compliance. It has been revealed that the increase in GH accelerates wound healing [28]. In a retrospective study, Sipahi and colleagues [29] found that the mixture of Gln/Arg/HMB for 4 weeks improves wound healing in diabetic patients. However, there was no difference between Gln/Arg/HMB and standard nutritional support (ensure) in the length of stay in the hospital. These contradictory results have been observed in other studies as well. In another study, supplementation of Gln/Arg/HMB did not affect the time of stay in ICU or hospital time of critically ill patients [30].

The combination of Gln/Arg/HMB may improve recovery through various mechanisms in patients undergoing heart surgery. This compound had beneficial effects on vascular endothelial function [31], intestinal injuries [32], pressure ulcers [20, 33], immune function [34], and anastomosis healing [35]. Moreover, it may prevent muscle wasting [36]. However, some studies failed to find the effect of Gln/Arg/HMB on muscle wasting [37–39]. Gln may be involved in the enhancement of the recovery through collagen deposition in the wound [40], the production of glutamic acid (which improves heart function and cardiac output) [41], reducing myocardial damage (indicated by the reduction in the troponin and CPK-MB) [14], preservation of intramyocardial glutamate, balancing the a-ketoglutarate and glutamate levels, helping to produce high-energy phosphates [42], and improving the immune function [43]. Arg may have beneficial effects through increasing blood flow, clearing the inflammation from the site of injury, deposition of collagen, and improving vascular endothelial function [15, 40]. HMB has several functions including accumulation of collagen in the wound, stimulating protein synthesis, preventing protein degradation, and suppression of inflammation [17, 40, 44]. Despite this evidence, the observation of conflicting findings may be due to the short duration of the intervention, the time of onset of the intervention, the small sample size, and the different physiological conditions between the diseases.

In the present study, the AST level was increased and the total and indirect bilirubin levels were decreased following Gln/Arg/HMB supplementation. Heart surgery may disturb the blood flow of different organs, including the gastrointestinal system, which leads to a 50% decrease in blood circulation and oxygen transport to the liver [45]. After surgery, factors including drugs that were used for anesthesia, heart failure, and blood flow disturbance lead to liver failure in 2.3% of patients [46, 47]. Also, postoperative jaundice causes 25% of deaths after open-heart surgery [48]. The damage to the hepatocyte, characterized by an increase in liver enzymes and hyperbilirubinemia, postpones the recovery process and prolongs the residence in ICU [49]. There are hypotheses about the mechanism of action of these amino acids. Previous studies showed that Gln may protect hepatocytes by preserving liver glutathione and enhancing antioxidant capacity [50]. Arg regulates the expression of inducible nitric oxide synthase (iNOS), stimulates the production of NO, increases the blood flow to the liver, and decreases liver injury [51]. HMB may be involved in preventing protein degradation in hepatic cells and eventually have positive effects on liver regeneration [52]. However, these hypotheses should be confirmed in clinical studies. In the study of Sipahi et al. [29], a significant increase was observed in ALT levels following treatment with a combination of Gln/Arg/HMB in diabetic patients. Erdem et al. [27] found no change in the serum ALT and AST levels after supplementation with Gln/Arg/HMB or standard nutrition support in patients with major burn. Considering scarce studies in this area, no definite conclusion can be made about the effectiveness of this compound on liver damage. Further clinical trials with enough sample size and duration are necessary to clarify this relationship.

To the best of our knowledge, no study has been published on the association between preoperative supplementation of Gln/Arg/HMB and postoperative recovery of cardiac surgery patients. This is the first randomized clinical trial in this regard. Also, the sample size of previous studies on other medical conditions was small, and most studies were conducted in short-term peri-operation. Therefore, the appropriate duration of intervention and relatively large sample size are among the strengths of the study. However, some potential limitations should be noted. Due to the coincidence with the COVID-19 pandemic, some individuals discontinued the study. The limitation in time and budget made it impossible to assess the plasma levels of amino acids, especially glutamine, to determine compliance. Moreover, in future studies, it is better to assess the baseline nutritional status and stratify participants accordingly.

## Conclusion

The present study showed that perioperative supplementation with a combination of Gln, Arg, and HMB enhances the recovery, reduces myocardial injury, and decreases the time of hospital and ICU stay in cardiac surgery patients. However, in some variables including LVEF, nausea, vomiting, nutritional risk, and complications after surgery, no change was observed. In addition, contradictory findings were observed regarding serum hepatic enzymes and bilirubin levels. Since this is the first study evaluating this combination on cardiac surgery outcomes, further studies are warranted to confirm these results and the mechanism of action.

## Data Availability

The datasets used and/or analyzed during the current study are available from the corresponding author on reasonable request.
